# Special Issue: Intramolecular Hydrogen Bonding

**DOI:** 10.3390/molecules191015783

**Published:** 2014-09-29

**Authors:** Ronald K. Castellano

**Affiliations:** Department of Chemistry, University of Florida, P.O. Box 117200, Gainesville, FL 32611-7200, USA; E-Mail: castellano@chem.ufl.edu; Tel.: +1-352-392-2752; Fax: +1-352-846-0296

**Keywords:** conformation, cooperativity, H-bonding, host-guest chemistry, non-covalent interactions, resonance-assisted hydrogen bonds, self-assembly, supramolecular chemistry, tautomerism, three-center hydrogen bonds

## Abstract

Intramolecular hydrogen bonds play critical structure- and function-serving roles in biological and synthetic molecular systems. This special issue, through eight contributions, showcases the prominence of these non-covalent interactions within several scientific disciplines, and in various structural contexts and environments. Reported, for example, are the consequences of intramolecular hydrogen bonds on the structures of molecules that show biological activity, for biological mechanisms, and for the conformational switching of functional synthetic molecules. Also showcased in the contributions are the state-of-the-art experimental and theoretical methods available for the characterization of intramolecular hydrogen bonds, which critically report on their strengths, geometries, and spectroscopic signatures in the gas, solid, and solution phases.

Hydrogen bonds are arguably the champion non-covalent interactions due to their intermediate (but tunable) strengths and predictable geometries. When formed from a hydrogen bond donor and acceptor within the same molecule, the resultant “intramolecular” hydrogen bond is uniquely capable of stabilizing conformation. Since conformation and function are intimately linked for many molecules, including most natural biomolecules, the importance of intramolecular hydrogen bonds is self-evident. In other contexts, including π-conjugated molecules, intramolecular hydrogen bonds participate in photophysical and photochemical processes. The generally enhanced strengths of intramolecular (versus intermolecular) hydrogen bonds allow them to persevere in the presence of other non-covalent interactions, in competitive solvent environments, and in the solid state. While fundamentally well understood, there remains no shortage of opportunities for either characterizing the interactions in functional contexts or deploying them as structure-promoting elements in designed molecular systems. Leveraging these efforts over the years, of course, is a host of technological advances that allow hydrogen bonds to be interrogated spectroscopically, structurally, and through quantum chemical calculations.

Contributions were welcomed for inclusion into this Special Issue of Molecules spanning all aspects of intramolecular hydrogen bonding, from theory to experiment, and from physical characterization to synthetic application. Eight excellent articles have been received that showcase the diverse structural and functional roles of intramolecular hydrogen bonds in different molecular contexts.

At the interface of molecular structure and biological function come four contributions. Martínez-Cifuentes and coworkers use theoretical and ^1^H-NMR analysis to fundamentally study the intramolecular C=O…H–O interactions in a family of *O*-carbonyl hydroquinones, some members of which have displayed anticancer and antioxidant activity. Showing a similar hydrogen bonding pattern and compelling biological activity are curcumin derivatives (e.g., cyclovalone), for which Nardo and coworkers derive relationships between H-bond structure and photochemical and photosensitization behavior. Radošević, Barišić, and coworkers have studied (by IR, NMR, CD, and X-ray) the conformational stabilization of small ferrocene–proline conjugates and gone on to preliminarily test their biological activity against breast and cervical cancer cell lines. Finally, Takahashi and coworkers use DFT calculations to propose a compelling single reaction model for the conversion of aspartic acid residues to succinimide, on the way to the amino acid’s isomerization and/or racemization, by way of a cyclic array of stabilizing hydrogen bonds.

Intramolecular hydrogen bonds are mainstay interactions for synthetic chemists interested in the preparation of functional molecules. The closed forms of the molecular baskets of Badjić and coworkers are stabilized by intramolecular hydrogen bonds that mediate encapsulation processes. In their contribution, the authors use NMR spectroscopy and molecular modeling to shed new light on the solvent/guest exchange dynamics and mechanism for one of their molecular hosts. Hamilton and coworkers have cleverly developed a molecule capable of redox-mediated conformational switching; key to the molecular function is modulation of intramolecular hydrogen bond strength.

Our ability to rationally use intramolecular hydrogen bonds is ultimately limited by our ability to characterize and fundamentally understand the interactions. Along these lines, Martínez-Martínez and coworkers have evaluated the energetics of three-center hydrogen bonds in model systems and shown how the interactions can be sufficient to overcome steric preferences in the solid state. Lastly, the contribution of Gerothanassis and coworkers collates the experimentally and theoretically derived descriptors for intramolecular hydrogen bonds persistent in families of phenol-containing natural products and model compounds.

The editors and I feel that we have only scratched the surface with this Special Issue; consequently, we will be looking for additional contributions next year. At that time, we will hope to receive submissions that not only expand the research themes highlighted in this issue, but also describe the roles of intramolecular hydrogen bonding in catalyst design and function, modulating photophysical and electronic structure, and dictating the conformations (and therefore the photochromic, thermochromic, or sensing behavior) of functional materials.

For now, enjoy this Special Issue on Intramolecular Hydrogen Bonding and join us in thanking all of the authors for their timely contributions.

